# No influenza D virus detected among pigs, northern Vietnam

**DOI:** 10.1111/irv.12812

**Published:** 2020-09-21

**Authors:** Amanda S. Farrell, Vuong N. Bui, Tung D. Dao, Trung D. Hoang, Gregory C. Gray

**Affiliations:** ^1^ Division of Infectious Diseases School of Medicine Duke University Durham NC USA; ^2^ Duke Global Health Institute Duke University Durham NC USA; ^3^ Vietnam National Institute of Veterinary Research Hanoi Viet Nam; ^4^ Global Health Research Center Duke Kunshan University Kunshan China; ^5^ Program in Emerging Infectious Diseases Duke‐NUS Medical School Singapore Singapore

1

Dear Editor Cowling,

In 2011, influenza D virus (IDV) was first isolated from US pigs that were exhibiting influenza‐like illness.^1^, ^2^ Though it was initially classified as a subtype of influenza C virus, IDV is now recognized as a novel member of the Orthomyxoviridae family of viruses.^3^ Since its discovery in pigs, IDV has been isolated in a number of other animal species including cattle, horses, sheep, goat, and camelids. Most recently, we found evidence of IDV in poultry in Sarawak, Malaysia.^4^

The zoonotic potential of IDV has not been extensively studied, and there are currently no known cases of IDV transmission among humans. Interestingly however, a 2015 study confirmed that bovine IDV can be replicated and transmitted among guinea pigs and ferrets, which are a model for human influenza virus infection.^5^ Additionally, in 2016, a cross‐sectional serological study conducted in Florida detected a marked increase in anti‐IDV antibodies among cattle workers compared to non‐cattle‐exposed individuals.^6^ Together, these studies suggest that IDV could be an emerging zoonotic threat.

Globally, IDV has also been identified in China,^7^ Japan,^8^ France,^9^ Italy,^10^ Argentina,^11^ Turkey,^12^ Kenya, Morocco, Togo, and Benin.^13^ A high density of animal farms and markets, limited public health resources, and variation in biosecurity measures make Vietnam an ideal location for the propagation of novel zoonoses like IDV. However, currently we know of no published literature on IDV surveillance among domesticated animals in Vietnam. As such, the primary aim of this investigation was to determine the prevalence of IDV among pigs in northern Vietnam.

**FIGURE 1:**
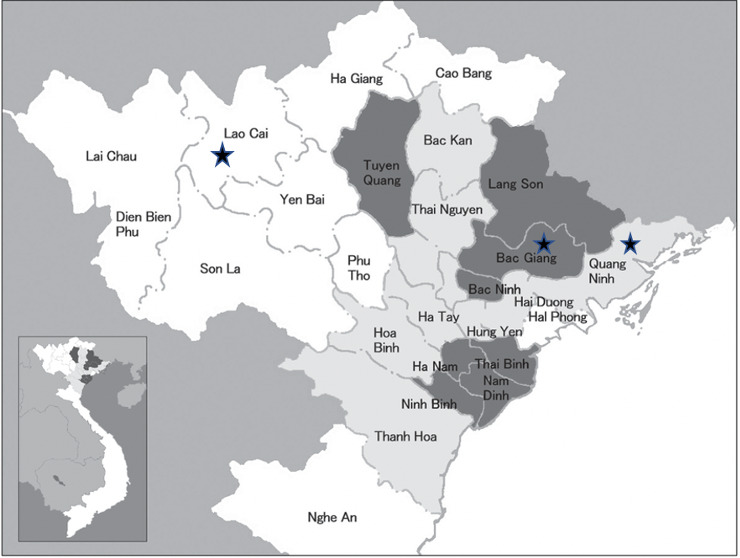
Geographic distribution of sampling locations (black stars). Samples were collected between May 2019 and February 2020 from the two farms in the Lao Cai province, two in Bac Giang, and one in Quang Ninh

From May 2019 to February 2020, as part of an ongoing influenza A virus surveillance study, we collected samples from five swine farms across northern Vietnam (Figure 1). The resultant 823 samples included bioaerosol samples (91, 11%), fecal samples (272, 33%), swine oral secretions (276, 34%), and farmworker nasal washes (184, 22%). The bioaerosol samples were collected using National Institute of Biosafety and Health's (NIOSH) model BC251 two‐stage bioaerosol samplers. The samplers were affixed to a stationary tripod, calibrated to a rate of 3.5 L/min, and placed in pig pens for 3 hours. FLOQSwabs (Copan Diagnostics) were used to collect the fecal samples from pig enclosures. Swine oral secretions were collected by fixing cotton ropes at animal height for chewing. Once the ropes were sufficiently chewed—for a period of approximately 30‐45 minutes—the fluid was extracted from the rope into sterile cryovials. Additionally, after appropriate consent, nasal washes were collected from farm employees by a trained assistant who injected 5 mL of sterile water into one nostril and collected the expressed fluid in a sterile specimen cup. All samples were labeled appropriately and stored at −80°C until RNA extraction was performed.

QIAamp Viral RNA Mini Kits (Qiagen) were used for RNA extraction. Viral RNA extracts were analyzed via quantitative real‐time polymerase chain reaction (qRT‐PCR) using Superscript III One‐Step RT‐PCR System with platinum (Thermo Fisher Scientific, Inc) and influenza D virus‐specific primers and probes.^1^ Synthetic positive and negative controls were used in each PCR run.

Influenza D virus was not detected in any of the samples. As prevalence among pigs has been high in other countries, this finding was unexpected. This might be explained in several ways. Perhaps there is no influenza D in these farms. We have also had difficulty detecting influenza A in these farms but, using the same assays and laboratorians, found a high prevalence of influenza A among live bird markets in the same geographical areas.^14^ It is also possible that there was an inherently low prevalence of IDV on the selected farms, most of which were confined to northern areas, larger and industrialized with solid biosecurity protocols in place. The inclusion of more farms, especially smaller, community farms, in other provinces might reverse our molecular influenza A observations.

Despite the limitations of geographical area and scale, our study is valuable as it is the first to assess IDV prevalence in Vietnam. A major strength of our study is the use of bioaerosol sampling technique which has been success for a number of viral surveillance studies. Future epidemiological investigations should be done to further characterize the prevalence of IDV in other regions of Vietnam and in other countries. Such studies of IDV will be essential for our understanding of its zoonotic potential and could impact biosecurity measures, such as use of personal protective equipment, on animal farms.

### CONFLICT OF INTEREST

None declared.

### AUTHOR CONTRIBUTIONS

**Amanda S Farrell:** Conceptualization (equal); Investigation (equal); Writing‐original draft (lead); Writing‐review & editing (supporting). **Vuong Nghia Bui:** Conceptualization (equal); Investigation (supporting); Methodology (supporting); Project administration (equal); Resources (equal); Supervision (lead); Writing‐review & editing (supporting). **Tung Duy Dao:** Investigation (equal); Methodology (supporting); Project administration (equal); Supervision (supporting); Writing‐review & editing (supporting). **Trung Duc Hoang:** Investigation (equal); Methodology (supporting). **Gregory C. Gray:** Conceptualization (equal); Methodology (lead); Project administration (equal); Supervision (supporting); Writing‐review & editing (lead).

### FUNDING INFORMATION

This study was supported by Professor Gray's discretionary funding and by funding from the Duke Global Health Institute, the Eugene A. Stead Student Research Fellowship, the American Society of Tropical Medicine and Hygiene, and the Infectious Disease Society of America.

REFERENCES1

Hause
BM
, 
Ducatez
M
, 
Collin
EA
, et al. Isolation of a novel swine influenza virus from Oklahoma in 2011 which is distantly related to human influenza C viruses. PLoS Pathog. 2013;9(2):e1003176.2340889310.1371/journal.ppat.1003176PMC35671772

Hause
BM
, 
Collin
EA
, 
Liu
R
, et al. Characterization of a novel influenza virus in cattle and Swine: proposal for a new genus in the Orthomyxoviridae family. MBio. 2014;5(2):e00031.2459536910.1128/mBio.00031-14PMC39587973

Ferguson
L
, 
Eckard
L
, 
Epperson
WB
, et al. Influenza D virus infection in Mississippi beef cattle. Virology. 2015;486:28‐34.2638655410.1016/j.virol.2015.08.030PMC47101784

Bailey
ES
, 
Fieldhouse
JK
, 
Alarja
NA
, et al. First sequence of influenza D virus identified in poultry farm bioaerosols in Sarawak, Malaysia. Trop Dis Travel Med Vaccines. 2020;6(1). 10.1186/s40794-020-0105-9
PMC7069008321903465

Sreenivasan
C
, 
Thomas
M
, 
Sheng
Z
, et al. Replication and transmission of the novel bovine influenza D virus in a guinea pig model. J Virol. 2015;89(23):11990‐12001.2637816110.1128/JVI.01630-15PMC46453316

White
SK
, 
Ma
W
, 
McDaniel
CJ
, 
Gray
GC
, 
Lednicky
JA
. Serologic evidence of exposure to influenza D virus among persons with occupational contact with cattle. J Clin Virol. 2016;81:31‐33.2729467210.1016/j.jcv.2016.05.0177

Zhai
S‐L
, 
Zhang
H
, 
Chen
S‐N
, et al. Influenza D Virus in animal species in Guangdong Province, Southern China. Emerg Infect Dis. 2017;23(8):1392‐1396.2872660910.3201/eid2308.170059PMC55478038

Murakami
S
, 
Endoh
M
, 
Kobayashi
T
, et al. Influenza D virus infection in herd of Cattle, Japan. Emerging Infect Dis. 2016;22(8):1517‐1519.10.3201/eid2208.160362PMC4982187274342139

Ducatez
MF
, 
Pelletier
C
, 
Meyer
G
. Influenza D virus in Cattle, France, 2011–2014. Emerg Infect Dis. 2015;21(2):368‐371.2562803810.3201/eid2102.141449PMC431366110

Chiapponi
C
, 
Faccini
S
, 
De Mattia
A
, et al. Detection of influenza D virus among Swine and Cattle, Italy. Emerg Infect Dis. 2016;22(2):352‐354.2681228210.3201/eid2202.151439PMC473454411

Alvarez
IJ
, 
Fort
M
, 
Pasucci
J
, et al. Seroprevalence of influenza D virus in bulls in Argentina. J Vet Diagn Invest. 2020;32(4):585‐588.3255251610.1177/1040638720934056PMC743865012

Yilmaz
A
, 
Umar
S
, 
Turan
N
, et al. First report of influenza D virus infection in Turkish cattle with respiratory disease. Res Vet Sci. 2020;130:98‐102.3216981110.1016/j.rvsc.2020.02.01713

Salem
E
, 
Cook
EAJ
, 
Lbacha
HA
, et al. Serological evidence for influenza C and D virus among ruminants and Camelids, Africa, 1991–2015. Emerg Infect Dis. 2017;23(9):1556‐1559.2882037110.3201/eid2309.170342PMC557287514

Bui
VN
, 
Nguyen
TT
, 
Nguyen‐Viet
H
, et al. Bioaerosol sampling to detect avian influenza virus in Hanoi’s largest live poultry market. Clin Infect Dis. 2019;68:972‐975.3018411410.1093/cid/ciy583
